# Radioablation +/− hormonotherapy for prostate cancer oligorecurrences (Radiosa trial): potential of imaging and biology (AIRC IG-22159)

**DOI:** 10.1186/s12885-019-6117-z

**Published:** 2019-09-10

**Authors:** Giulia Marvaso, Delia Ciardo, Giulia Corrao, Sara Gandini, Cristiana Fodor, Dario Zerini, Damaris Patricia Rojas, Matteo Augugliaro, Giuseppina Bonizzi, Salvatore Pece, Federica Cattani, Ketti Mazzocco, Francesco Alessandro Mistretta, Gennaro Musi, Sarah Alessi, Giuseppe Petralia, Gabriella Pravettoni, Ottavio De Cobelli, Pier Paolo Di Fiore, Giuseppe Viale, Roberto Orecchia, Barbara Alicja Jereczek-Fossa

**Affiliations:** 1 Division of Radiation Oncology, IEO, European Institute of Oncology IRCCS, Via Ripamonti 435, 20141 Milan, Italy; 2 Department of Oncology and Hematoncology, University of Milan, Milan, Italy; 3 Department of Experimental Oncology, IEO, European Institute of Oncology IRCCS, Milan, Italy; 4 Molecular Medicine Program, IEO, European Institute of Oncology IRCCS, Milan, Italy; 5 Unit of Medical Physics, IEO, European Institute of Oncology IRCCS, Milan, Italy; 6 Applied Research Division for Cognitive and Psychological Science, IEO, European Institute of Oncology IRCCS, Milan, Italy; 7 Department of Urology, IEO, European Institute of Oncology IRCCS, Milan, Italy; 8 Division of Radiology, IEO, European Institute of Oncology IRCCS, Milan, Italy; 9 Department of Pathology, IEO, European Institute of Oncology IRCCS & State University of Milan, Milan, Italy; 10 Scientific Direction, IEO, European Institute of Oncology IRCCS, Milan, Italy

**Keywords:** Prostate cancer, Radiotherapy, Oligometastasis, Androgen deprivation therapy, Stereotatic body radiation therapy

## Abstract

**Background:**

Prostate cancer (PCa) is the second most common cancer among men. New imaging-modalities have increased the diagnosed patients with limited number of metastasis after primary curative therapy, introducing so-called oligometastatic state. Stereotactic body radiotherapy (SBRT) is emerging as a low-toxicity treatment to erase PCa localizations and postpone androgen deprivation therapy (ADT). A deeper understanding of the predictive role of biomarkers is desirable for a targeted treatment selection and surveillance programs. The aims of the RADIOSA trial are:

1. Compare SBRT +/− ADT for oligorecurrent-castration-sensitive PCa (OCS-PCa) in terms of efficacy, toxicity and Quality of Life (QoL).
2. Develop biology/imaging based prognostic tool that allows identifying OCS-PCa subclasses.

**Methods:**

This is a randomized phase II clinical trial, recruiting 160 OCS-PCa in 3 years, with progression-free survival (PFS) as primary endpoint. Three tasks will be developed:

1. Randomized clinical study (3 years for accrual and 2 years for follow-up and data analysis);
2. Imaging study, including imaging registration and METastasis Reporting and Data System (MET-RADS) criteria;
3. Pre-clinical study, development of a biobank of blood samples for the analysis of neutrophil-to-lymphocyte ratio and preparatory for a subsequent miRNA profiling.

We aim to determine which arm is justified for testing in a subsequent Phase III trial. A decision-tree algorithm, based on prognosis, biological phenotype and imaging profile, will be developed.

**Discussion:**

Recruiting will start in July 2019. SBRT will allow obtaining excellent PFS, local control, QoL and low toxicity. In SBRT arm, ADT deferral will allow for a drug-holiday, delaying the detrimental impact on QoL. A sufficient number of blood samples will be collected to perform biological patient profiling. A stratification tool will be established with an analysis of morphological and functional imaging, based on the use of MET-RADS criteria.

So, in conclusion, RADIOSA aims to define the optimal management of bone/nodal PCa relapses in a SBRT regimen. This study will increase our knowledge on low-burden metastatic PCa in the era of high precision and high technology personalized medicine, offering highly effective therapy in terms of clinical outcome and cost-effectiveness.

**Trial registration:**

The RADIOSA study was prospectively registered at clinicaltrials.gov (NCT03940235, May 2019).

## Background

PCa is the second most common cancer among men in the world (1.1 million new cases estimated) and death PCa related (estimated in over 300,000 deaths annually) correlates with metastatic state [1].

The advent of novel imaging techniques, such as Prostate-Specific Membrane Antigen Positron Emission Tomography (PSMA PET) and Whole-Body Magnetic Resonance Imaging (WB MRI), allows an increasing an early detection and diagnosis of metastases after primary curative treatment. In clinical practice the possibility to identify a few number of lesions is called “oligometastatic state” and it is considered an intermediate phase of tumor spread with limited metastatic capacity [2]. This state consists of low-volume disease, typically defined as the presence of up to 3–5 metastases [3] treatable with a local approach (such as surgery or radiotherapy) either with or without systemic therapy.

Patients with low-volume or oligometastatic disease have improved survival compared with those with high-volume metastases or a disseminated metastatic cancer [4]. While chemo-hormonal therapy is the standard of care for men with high-volume metastatic castration-sensitive PCa [5], in the low-volume metastatic PCa additional chemo-therapy did not demonstrate survival benefit [6], so SBRT is emerging as a promising low-toxicity treatment option for oligometastases both at diagnosis and recurrence [7]. Small-volume high-dose SBRT limited to metastatic site might potentially eliminate all macroscopic cancer foci prolonging the progression-free interval and postponing ADT. The aim of SBRT in this setting is to achieve local control and delay progression, and thereby postpone the need for further treatment.

Moreover, an open question in this framework is the patient QoL, to be evaluated in the balance of oligometastatic disease diagnosis, less treatment option and additional ADT-induced side effects. A deeper understanding of the predictive role of biomarkers is desirable for a targeted treatment selection and proper surveillance programs. The limitation associated with the use of the prostate-specific antigen (PSA) as diagnostic and follow-up marker stimulated significant investigations of several novel biological markers, such as blood-based parameters, microRNAs and cell-free DNA [8, 9]. However, this new approach requires a large amount of coherently collected data to give reliable and univocal results.

A further research frontier is the role of imaging in this setting of patients. Recently, the role of WB MRI has been proposed as a suitable solution for tumor detection and therapy evaluation in this setting of patients with performance at least comparable with the Choline positron emission tomography/computed tomography Ch-PET/CT [10].

Overall, clear-cut data and tools to guide a consensus about the optimal therapeutic choice for the individual clinical situation are not validated and the standard of care to recurrent PCa remains ADT, with significant side effect and deterioration of QoL [11].

## Methods/design

### Aim, design, and setting of the study

The present research project aims to define the optimal management of bone or nodal PCa recurrences in a SBRT regimen. As a final result, a decision-tree algorithm, based on prognosis, biological phenotype and imaging profile, will be obtained as a clinically useful tool.

This study will move forward our knowledge on low-burden metastatic PCa in the era of high-precision and high technology personalized medicine, offering highly effective therapy in terms of clinical outcome and cost effectiveness. The aim is to compare time to progression between the two study arms: SBRT only or SBRT and ADT. RADIOSA will be developed during the 5 years of the project and it is divided in two main phases.

### Study population

#### Participant characteristics

RADIOSA is a prospective monocentric randomized phase II study in which oligorecurrent PCa patients (1–3 lesions) are randomized to two treatments and the results with the longest time to progression is selected for further study [12].

#### Eligibility criteria

The inclusion criteria are as follows:

1. Histologically proven initial diagnosis of adenocarcinoma of the prostate
2. Biochemical relapse of PCa following radical local prostate treatment (radical prostatectomy, primary radiotherapy or radical prostatectomy +/− prostate bed adjuvant/salvage radiotherapy) +/− ADT according to the European Association of Urology (EAU) guidelines 2016 [13] or after any salvage therapy if biochemical progression is diagnosed in the context of castration sensitive PCa
3. Nodal relapse in the pelvis, extra-regional nodal relapse (M1a), bone metastases (M1b) on Ch-PET/CT or WB MRI with a maximum of 3 lesions
4. Serum testosterone level > 50 ng/dl at the time of randomization (castration sensitive PCa)
5. Eastern Cooperative Oncology Group (ECOG) performance status 0–1
6. Age ≥ 18 years
7. Ability to complete questionnaires about QoL
8. Written informed consent signed.

The exclusion criteria are as follows:

1. Presence of visceral metastases
2. More than three metastases
3. Inability to complete questionnaires about QoL
4. Previous invasive cancer (within 3 years before the prostate cancer diagnosis) apart from non-melanoma skin malignancies;
5. Serious concomitant comorbidities or contraindication to SBRT and/or ADT
6. Mental diseases that cannot ensure valid informed consent

#### Methods of recruitment and random allocation

In phase 1 a hundred sixty consecutive patients will be enrolled at the Division of Radiation Oncology at the European Institute of Oncology (IEO), Milan, Italy within a multidisciplinary uro-oncology board.

**Fig. 1:**
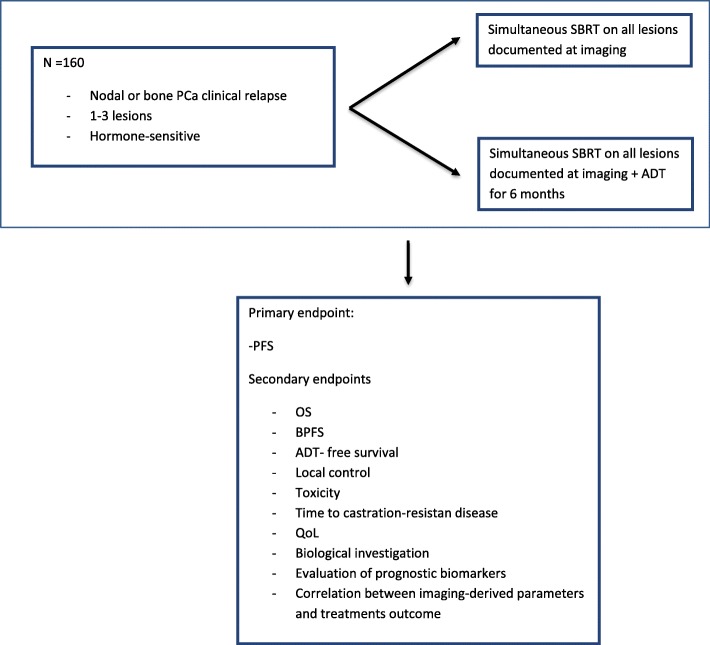
Study design

There will be a 1:1 randomization between Arm 1 and Arm 2. Patients will be stratified according to PSA doubling time (≤3 vs. > 3 months) [14, 15], initial localization of metastases (node vs bone) and diagnostic imaging (PET vs. MRI) [Fig. 1].

The patients will be randomized in either:

- ARM 1: salvage SBRT for lymph nodes and/or bone metastases. All the radiologically documented lesions will be treated simultaneously.
- ARM 2: salvage SBRT (as described for ARM 1) + 6-month ADT (luteinizing hormone-releasing hormone (LHRH) agonist or antagonist). ADT should start within 1 week before the start of SBRT.

Phase 2 is complementary to Phase 1. After SBRT completion, patients will be followed-up with PSA and testosterone measurements every 3 months and with clinical examination at 3 and 6 months and every 6 months thereafter. Questionnaires for the assessment of QoL will be administered within 2 weeks prior to randomization, at 3 and 12 months, and yearly thereafter.

Blood and plasma samples for biological evaluation and randomization of patients, at 3 months from completion of treatment and at clinical relapse. The samples will be stored in the “European Institute of Oncology Biobank”.

Acute and late toxicity will be assessed according to the Common Toxicity Criteria for adverse events (CTCAE) toxicity criteria v4.3 [16], evaluated at 1 (acute toxicity) and at 3–6 months after the end SBRT and yearly afterwards (late toxicity). Time to castration-resistant disease will be assessed as time to start a systemic therapy (testosterone evaluation). A timeline of this study is summarized in Table 1.

**Table 1:** Timeline of the study

	Screening	2 weeks within randomization	1 months after end of RT	3 months after end of RT	Every 3 months	12 months after end of RT	Every year	Biochimical relapse	Clinical relapse
Physical examination	x			x		x			
PSA/testosterone	x			x	x	x			
GS	x								
Previous treatments for PCa	x								
ECOG score	x								
Comorbidities	x								
Imaging	x								
Blood tests (NLR)		x							
Serum and plasma collection		x		x					x
Acute toxicity assessment			x						
Late toxicity assessment				x					
QoL		x		x		x	x		x
Cho-PET/TC or RM-WB								x	x

Moreover, a decision-tree algorithm, based on prognosis, imaging profile and biological phenotype will be developed: it will be addressed to identify recurrent PCa that remains oligometastatic and those that rapidly progress to a polymetastatic phase, thus allowing in the future to stratify patients who might avoid, or at least delay ADT, or not.

Data will be published at the end of the end of enrollment (2024).

#### Treatment

Within 35 days prior to randomization, either a WB MRI or a Ch-PET/CT is mandatory to confirm a diagnosis of oligorecurrent disease, with a maximum of 3 pelvic/extra-pelvic lymph nodes and/or bone metastases, and to exclude visceral disease.

According to SBRT technique, high doses will be delivered to small volumes in few fractions (≤5). In our framework, a schedule of 30 Gy in 3 fractions every other day (equivalent dose EQD2 = 98.6 Gy, considering α/β = 1.5 Gy), or equivalent regimens depending on disease site location will be used. Treatment planning and delivery will be performed using dedicated systems, such as Iplannet-Vero (Brainlab-Mitsubishi) and Multiplan-CyberKnife (Accuray), Eclipse-Trilogy (Varian) and Tomotherapy (Accuray). As far as image-guidance is concerned, Vero, Trilogy and Tomotherapy use a pre-treatment cone-beam CT whereas CyberKnife is based on intrafraction X-Rays. Thus, CyberKnife needs fiducials or spine tracking. CyberKnife is preferred when particular attention to critical structures is required and spine tracking is possible. Strict quality assurance protocols will be implemented to ensure the accuracy in dose delivery.

#### Endpoints of the study

The primary objective is to compare the progression-free survival (PFS) defined as the absence of new metastatic lesions (local, regional or distant) between the two arms.

The secondary endpoints are as follows:

1. comparison of overall survival (OS)
2. biochemical progression-free survival (BPFS)
3. ADT-free survival
4. local control
5. treatment-induced acute and late toxicity
6. time to castration-resistant disease
7. QoL between the two arms
8. development of a dedicated biobanking (collection of plasma and serum) for further biological investigation of predictive/diagnostic factors for personalized treatment
9. the preliminary evaluation of prognostic biomarkers
10. the correlation between imaging-derived parameters and treatment outcome

The present research project will be developed for 60 months, during which specific milestones will be achieved and documented.

#### Discontinuation of the treatment/ disease progression

In case of biochemical progression (defined according to the EAU guidelines [13]) after SBRT, a new imaging scan should be considered: a WB MRI or Ch-PET/CT scan at time of biochemical progression or symptomatic progression and 6-montly afterwards until clinical progression will be performed. Further treatments (including ADT or other treatments according to the institutional policy) should not be started for biochemical progression without documented clinical progression on WB MRI or Ch-PET/CT. Repeated SBRT is allowed.

#### Ethical consideration and study registration

The study will be performed in accordance with the Declaration of Helsinki and will comply to the International Conference on Harmonization and Good Clinical Practice. All possible treatments and examinations for CRPC are undertaken after obtaining written informed consent from the patients before registration. This trial was supported by the research grants from the Italian Association for Cancer Research (AIRC) IG-22159.

The RADIOSA study was approved by the Ethics Committee of “IRCCS Istituto Europeo di Oncologia and Centro Cardiologico Monzino” (IEO-997).

The study has been registered at clinicaltrials.gov (NCT03940235).

#### Calculation of the target sample size

The main endpoint of this study is time to progression (local, biochemical and clinical progression) in oligometastatic PCA patients. We designed a randomized phase II selection study in which patients are randomized to two treatments and the results with the longest time to progression is selected for further study [12]. The aim is to compare time to progression between the two study arms: SBRT only or SBRT and hormonotherapy, with α = 0.10 and β = 0.20. It is estimated that the median delay to start palliative ADT after metastasis-directed therapy is approximately 24 months [17]. In order to detect a 12-month difference in the main endpoint from 12 to 24 months, a total of 152 patients will be needed, using a two-sided hypothesis based on the F distribution. Assuming a 5% rate of loss to follow-up, a total of 160 patients will be accrued over 36 months with 24 months of additional follow-up. We expect an accrual rate of 53 patients per year.

## Discussion

Recently, the concept of ADT-free survival, intended as the time to the delayed start of ADT, in order to spare the related negative side effect, such as the increased occurrence of cardiovascular events and metabolic syndrome, that significantly affect the QoL [7]. The aim of SBRT in this setting is to achieve local control and delay progression, and thereby postpone the need for further treatments.

From the clinical standpoint, the emergence of castration resistance is usually a nearby antecedent of widespread disease progression, visceral involvement and death from PCa. Therefore, preservation of the responsiveness to ADT is crucial in the long-term management of patients with metastatic PCa.

Also, from socio-economic point of view, 12 months without ADT can be considered a consistent gain for the National Health System, especially considering the relevant costs of the new and spreading anti-androgens like Abiraterone and Enzalutamide [5].

Current knowledge is mainly based on retrospective and non-randomized studies, thus suffering from heterogeneous population (i.e., due to biased patient selection) and inappropriate sample power [18].

It is evident that also in the setting of metastatic PCa, prostatic definitive radiotherapy could find a place. First data derived from retrospective trials by Löppenberg et al. and Rusthoven et al. demonstrated an improvement in OS for patients who underwent radiation therapy targeting the prostate in comparison to those who received only hormonal treatment [19, 20].

The oligometastatic concept has been developed in the last years and recent trials show interesting results on adding local treatments (LCT) (such as radiotherapy or surgery) at standard therapy. Particularly, it is important to mention the results achieved by Gomez and Palma. Gomez et al., that conducted a randomized phase II trial on oligometastatic NSCLC, reporting a significant PFS benefit with LCT (both radiotherapy or surgery) vs maintenance systemic therapy (14.4 months vs 4.4 months respectively) [18]. In another randomized phase II trial, named SABR-COMET, a benefit in OS in the SBRT arm for the oligometastatic setting compared to palliative treatment was demonstrated (41 months vs 28 months) [21].

The HORRAD study is a multi-centric prospective study, randomized controlled trial recruiting bone metastatic PCa patients. The objective was to compare irradiation of primary prostatic tumor with external beam radiation therapy with ADT versus ADT only. The trial revealed no significant difference in OS. However, subgroup analysis suggests that radiotherapy to the prostate actually improves OS in patients with a low metastatic burden (< 5 bone lesions) [22].

More recently, subgroup analysis of patients with a low metastatic burden from STAMPEDE (a phase 3 randomized controlled trial) showed that OS was improved with radiotherapy, giving a three-year survival of 81% in these men, compared to 73% in the standard treatment group [5].

For Murphy et al., the approach to metastatic PCa “catching ‘em all”, or “pokemet”, must be considered experimental [23]. Also, in this setting, data from retrospective studies suggest that cancer specific and OS is improved with Metastasis-directed therapy (MDT) compared to the standard of care. MDT treatment regimens vary with different radiotherapy techniques, doses, and volumes [3]. Among prospective studies, STOMP and ORIOLE trials are worth mentioning [24, 25].

However, despite encouraging data, some authors remain skeptical that MDT will delay the use of ADT. Moreover, this argument is a weak endpoint compared to PFS or OS especially given that timing for using hormonotherapy remains controversial.

Whereas the use of systemic therapy was largely investigated by several large clinical trials, currently, limited studies focus on metastasis-directed SBRT. These studies, despite short follow-up that limited the evaluation of OS and cancer-specific survival, showed that metastasis-directed SBRT is a safe treatment, reaching a high local control (about 95%) with low-grade toxicity, generally limited to gastro-intestinal effects [3, 26–29].

So, new biomarkers are mandatory to help determining the natural history of the disease and to select the patients who could actually benefit from MDT. The discovery of miRNA seems to give a start of answers to many questions that arise.

Recent studies have demonstrated that miRNAs influence many stages of carcinogenesis and can effectively modulate tumor radiosensitivity at different levels, by affecting targets involved in DNA damage repair, cell cycle checkpoint, apoptosis, signal transduction pathways and tumor microenvironment. Thus, the identification of miRNA involved in DNA damage radiation response is not excluded and might lead to the use of Poly (ADP-ribose) polymerase (PARP) inhibitors in the near future [26].

In a recent published study by Pitroda PS et al., a molecular basis (such as mi-RNA profiling) for oligometastatic state in colorectal liver metastasis was identified in order to predict clinical outcome and establish clinical risk factors associated with long-term survival following hepatic resection [30].

According to Formosa et al. a series of miRNAs called oligomers would be typical of oligometastatic diseases, and thus have a role in the developmentof the oligometastatic state and the transition from an oligometastatic state to a polymetastatic disease [31].

Overall, data and tools to guide an optimal therapeutic choice in this setting are not validated and the standard of care to recurrent PCa remains ADT, with significant side effects and deterioration of QoL [10]. To address a larger consensus on oligorecurrent PCa management, randomized clinical trials selecting homogeneous patient population are needed.

## Data Availability

Data of this article will be not available until the final report of this study to avoid bias toward the analysis.

## Abbreviations

ADT: Androgen deprivation therapy
AIRC: Italian Association for Cancer Research
BPFS: Biochemical progression-free survival
Ch-PET/CT: Choline positron emission tomography/computed tomography
CTCAE: Common Toxicity Criteria for adverse events
EAU: Association of Urology
ECOG: Eastern Cooperative Oncology Group
IEO: European Institute of Oncology
LCT: Local treatments
LHRH: Luteinizing hormone-releasing hormone
MDT: Metastasis-directed therapy
MET-RADS: METastasis Reporting and Data System
OCS-PCa: Oligorecurrent-castration-sensitive PCa
OS: Overall survival
PARP: Poly (ADP-ribose) polymerase inhibitors
pCa: Prostate Cancer
PFS: Progression-free survival
PSA: Prostate-specific antigen
PSMA PET: Prostate-Specific Membrane Antigen Positron Emission Tomography
QoL: Quality of Life
SBRT: Stereotactic body radiotherapy
WB MRI: Whole-Body Magnetic Resonance Imaging
